# Atrial Fibrillation and Outcomes in Heart Failure With Preserved Versus Reduced Left Ventricular Ejection Fraction

**DOI:** 10.1161/JAHA.112.005694

**Published:** 2013-02-22

**Authors:** David D. McManus, Grace Hsu, Sue Hee Sung, Jane S. Saczynski, David H. Smith, David J. Magid, Jerry H. Gurwitz, Robert J. Goldberg, Alan S. Go, for the Cardiovascular Research Network PRESERVE Study

**Affiliations:** 1 Division of Cardiovascular Medicine, University of Massachusetts Medical School, Worcester, MA (D.D.M.M.); 2 Department of Quantitative Health Sciences, University of Massachusetts Medical School, Worcester, MA (D.D.M.M., J.S.S., J.H.G., R.J.G.); 3 Meyers Primary Care Institute, Fallon Community Health Plan, and Reliant Medical Group, University of Massachusetts Medical School, Worcester, MA (D.D.M.M., J.S.S., J.H.G., R.J.G.); 4 Department of Biomedical Engineering, Worcester Polytechnic Institute, Worcester, MA (D.D.M.M.); 5 Division of Research, Kaiser Permanente of Northern California, Oakland, CA (G.H., S.H.S., A.S.G.); 6 Departments of Epidemiology, Biostatistics, and Medicine, University of California, San Francisco, CA (G.H., S.H.S., A.S.G.); 7 Center for Health Research, Kaiser Permanente Northwest, Portland, OR (D.H.S.); 8 Institute for Health Research, Kaiser Permanente Colorado, Denver, CO (D.J.M.); 9 Department of Health Research and Policy, Stanford University School of Medicine, Palo Alto, CA (A.S.G.)

**Keywords:** atrial fibrillation, heart failure, hospitalization, mortality, systolic function

## Abstract

**Background:**

Atrial fibrillation (AF) and heart failure (HF) are 2 of the most common cardiovascular conditions nationally and AF frequently complicates HF. We examined how AF has impacts on adverse outcomes in HF‐PEF versus HF‐REF within a large, contemporary cohort.

**Methods and Results:**

We identified all adults diagnosed with HF‐PEF or HF‐REF based on hospital discharge and ambulatory visit diagnoses and relevant imaging results for 2005–2008 from 4 health plans in the Cardiovascular Research Network. Data on demographic features, diagnoses, procedures, outpatient pharmacy use, and laboratory results were ascertained from health plan databases. Hospitalizations for HF, stroke, and any reason were identified from hospital discharge and billing claims databases. Deaths were ascertained from health plan and state death files. Among 23 644 patients with HF, 11 429 (48.3%) had documented AF (9081 preexisting, 2348 incident). Compared with patients who did not have AF, patients with AF had higher adjusted rates of ischemic stroke (hazard ratio [HR] 2.47 for incident AF; HR 1.57 for preexisting AF), hospitalization for HF (HR 2.00 for incident AF; HR 1.22 for preexisting AF), all‐cause hospitalization (HR 1.45 for incident AF; HR 1.15 for preexisting AF), and death (incident AF HR 1.67; preexisting AF HR 1.13). The associations of AF with these outcomes were similar for HF‐PEF and HF‐REF, with the exception of ischemic stroke.

**Conclusions:**

AF is a potent risk factor for adverse outcomes in patients with HF‐PEF or HF‐REF. Effective interventions are needed to improve the prognosis of these high‐risk patients.

## Introduction

Heart failure (HF) and atrial fibrillation (AF) represent 2 worsening epidemics nationally and internationally.^1–3^ AF remains the most common, clinically relevant arrhythmia in adults and is independently associated with a 4‐ to 5‐fold higher risk of ischemic stroke, as well as poorer quality of life, higher hospitalization rates, and excess mortality.^4–6^ AF frequently complicates HF, affecting approximately one third of all adults with HF.^7^ However, the epidemiology of HF has been changing, with an increasing proportion of patients being diagnosed with HF and preserved left ventricular ejection fraction (HF‐PEF).^8^ Limited data exist on contemporary incidence rates and outcomes associated with AF in the setting of HF‐PEF compared with HF and reduced ejection fraction (HF‐REF).^9^

Within a large, multicenter cohort of adults with HF, we examined the association of preexisting versus incident AF with clinically relevant outcomes among adults with HF‐PEF and HF‐REF.

## Methods

### Source Population

The source population included members from 4 participating health plans within the National Heart, Lung, and Blood Institute–sponsored Cardiovascular Research Network (CVRN).^10^ Sites included Kaiser Permanente Northern California, Kaiser Permanente Colorado, Kaiser Permanente Northwest, and Fallon Community Health Plan. The sites were identified on the basis of providing care to an ethnically and socioeconomically diverse population across varying clinical practice settings and geographically diverse areas. Each site also had a Virtual Data Warehouse (VDW),^10^ which served as the primary data source for identifying and characterizing study subjects. The CVRN VDW is a distributed standardized data resource composed of linked demographic, pharmacy, laboratory test results, and health care utilization (outpatient visits as well as health plan and non–health plan hospitalizations with diagnoses and procedures) data for health plan members receiving care within participating CVRN sites.^11,10^

Institutional review boards at participating sites approved the study, and waiver of consent was obtained due to the nature of the study.

### Study Sample

We identified all individuals aged ≥21 years with diagnosed HF between January 1, 2005, through December 31, 2008, based on either ≥1 hospitalization with a primary discharge diagnosis of HF and/or ≥3 ambulatory visits coded for HF with ≥1 of the ambulatory diagnoses from a cardiologist to enhance diagnostic specificity. The following *International Classification of Diseases*,* Ninth Edition* (ICD‐9) codes were used to identify potential HF cases: 398.91, 402.01, 402.11, 402.91, 404.01, 404.03, 404.11, 404.13, 404.91, 404.93, 428.0, 428.1, 428.20, 428.21, 428.22, 428.23, 428.30, 428.31, 428.32, 428.33, 428.40, 428.41, 428.42, 428.43, and 428.9. When compared against medical record abstraction and use of the Framingham Heart Study clinical criteria, use of the primary discharge diagnosis of HF based on these codes showed a positive predictive value of >95%.^12–14^ We determined the level of left ventricular systolic function closest to the qualifying HF diagnosis, based on clinically obtained echocardiograms and other relevant imaging modalities. We classified patients into categories of preserved and reduced left ventricular ejection fraction. We defined PEF as left ventricular ejection fraction ≥50% and/or a physician's qualitative assessment of preserved or normal systolic function.^15^ REF was defined as left ventricular ejection fraction ≤40% and/or a physician's qualitative assessment of moderate, moderate‐to‐severe, or severe systolic dysfunction. To limit misclassification, we excluded participants with ejection fraction >40% and <50% and/or a physician's qualitative assessment of mild systolic dysfunction.

### Definition of AF

We ascertained AF based on ≥1 primary hospital discharge and/or ≥2 ambulatory diagnoses of AF (ICD‐9 code 427.31) or atrial flutter (ICD‐9 code 427.32) from each site's VDW.^4^ We defined preexisting AF as AF documented any time during the 5 years before cohort entry, and incident AF as AF occurring anytime during follow‐up among those patients with HF without AF at baseline.

### Follow‐up and Outcomes

Follow‐up occurred through December 31, 2008, which was the latest date on which complete data on death were available at the time of analysis. Subjects were censored if they disenrolled from the health plan or reached the end of study follow‐up. Hospitalizations for HF were identified from each site's VDW based on a primary discharge diagnosis for HF using the same inclusion criteria ICD‐9 codes. Ischemic strokes were identified from hospital discharge and billing claims databases using previously validated ICD‐9 codes.^16^ Occurrence of death was identified using data from member proxy report, state death certificate registries, and Social Security Administration files as available at each site. These approaches have yielded >97% vital status information in our prior studies.^13,17^

### Covariates

We ascertained information on coexisting illnesses based on diagnoses or procedures using relevant ICD‐9 codes, laboratory results, or filled outpatient prescriptions from health plan hospitalization discharge, ambulatory visit, laboratory, and pharmacy databases, as well as from site‐specific diabetes mellitus and cancer registries.^18^ We defined prevalent HF as any hospitalization or ambulatory HF diagnosis before the index date. We collected baseline and follow‐up data on diagnoses of coronary artery disease, acute myocardial infarction, coronary artery revascularization, stroke and transient ischemic attack, peripheral artery disease, diabetes, hypertension, cancer, liver disease, valvular heart disease, lung disease, and ventricular fibrillation/tachycardia based on previously described ICD‐9 codes and Current Procedural Terminology procedure codes.^18^ For the purposes of this study, “baseline” was defined by the period 5 years before the index date for data regarding comorbidities and laboratory values. For medication use, “baseline” was determined by any use within 120 days before the index date and active use within 30 days of index date.

Using each site's VDW, we captured ambulatory measurements of systolic and diastolic blood pressure, serum low‐density lipoprotein cholesterol, and blood hemoglobin level on or before the index date and during follow‐up. We also classified baseline and longitudinal kidney function using the Chronic Kidney Disease Epidemiology Collaboration formula for estimating glomerular filtration rate (mL/min per 1.73 m^2^) based on outpatient serum creatinine results.^19^ We characterized longitudinal receipt of HF‐related medications including angiotensin‐converting enzyme inhibitors, angiotensin receptor blockers, β‐blockers, digoxin, thiazide and loop diuretics, nitrates, aldosterone receptor antagonists, and statins using previously described methods.^12^ We also identified receipt of cardiac resynchronization therapy (with or without defibrillator), implantable‐cardioverter defibrillator placement, and pacemaker placement using ICD‐9 procedure and Current Procedural Terminology codes.^10^

### Data Analyses

All analyses were conducted using SAS statistical software, version 9.2 (SAS Institute). We compared baseline characteristics by AF status (none, preexisting AF, and incident AF) using the following methods: Kruskal–Wallis test for comparing median values, ANOVA for comparing mean values, and χ^2^ tests for comparing categorical variables. Because exclusion of the 8639 participants with rheumatic and aortic or mitral valve disease did not materially affect study findings, we included these participants in the main analysis. Although we considered a 2‐sided *P* value <0.05 as statistically significant, given the large sample size, we focused only on differences across groups that may be clinically meaningful.

We calculated rates (per 100 person‐years) and associated 95% CIs for each outcome according to AF status among patients with HF, overall and stratified by HF‐PEF versus HF‐REF using a time‐to‐event approach. We then conducted multivariable extended Cox regression models to examine the association between AF status and each outcome, overall and separately in those with HF‐PEF versus HF‐REF. Death was treated as a censoring event when analyzing time to event outcomes.

## Results

Among 23 644 adults with HF, 60% had confirmed HF‐PEF (mean age 74.2 years, 47.7% were women, and 76.1% were white). Overall, 9081 (38.4%) had preexisting AF and 2348 (9.9%) had developed newly diagnosed (incident) AF during the study period (Table 1). The frequencies of preexisting and incident AF were 43.2% and 9.5%, respectively, in those with HF‐PEF, and 31.4% and 10.5%, respectively, in participants with HF‐REF. As expected, there was a high burden of vascular and nonvascular morbidity at study entry in the overall cohort (Table 1**)**. Of note, at baseline, 58% of the overall cohort received an angiotensin‐converting enzyme inhibitor or angiotensin receptor blocker, 63% of participants received a β‐blocker, and 26% received a calcium channel blocker (Table 2).

**Table 1.** Baseline Characteristics Among 23 644 Adults With Heart Failure and Preserved or Reduced Left Ventricular Systolic Function Identified During 2005–2008, Overall and Stratified by AF Status

Variable	Overall (N=23 644)	No AF (N=12 215)	Preexisting AF (N=9081)	Incident AF (N=2348)	*P* Value
Age (y), mean (SD)	74.2 (12.1)	71.6 (13.1)	77.5 (10.2)	75.4 (10.6)	<0.001
Age categories, y					<0.001
<45	484 (2.0)	418 (3.4)	51 (0.6)	15 (0.6)	
45 to 64	4584 (19.4)	3185 (26.1)	1024 (11.3)	375 (16.0)	
65 to 74	5838 (24.7)	3172 (26.0)	2042 (22.5)	624 (26.6)	
≥75	12 738 (53.9)	5440 (44.5)	5964 (65.7)	1334 (56.8)	
Female sex, n (%)	11 283 (47.7)	5880 (48.1)	4362 (48.0)	1041 (44.3)	0.003
Race, n (%)					<0.001
White	17 985 (76.1)	8665 (70.9)	7489 (82.5)	1831 (78.0)	
Black/African American	1799 (7.6)	1260 (10.3)	356 (3.9)	183 (7.8)	
Asian	1194 (5.0)	681 (5.6)	406 (4.5)	107 (4.6)	
Native Hawaiian/other Pacific Islander	178 (0.8)	117 (1.0)	44 (0.5)	17 (0.7)	
Missing	2488 (10.5)	1492 (12.2)	786 (8.7)	210 (8.9)	
Clinical characteristics, n (%)					
Acute myocardial infraction	3080 (13.0)	1832 (15.0)	957 (10.5)	291 (12.4)	<0.001
Unstable angina	1630 (6.9)	916 (7.5)	566 (6.2)	148 (6.3)	0.001
Coronary artery bypass graft surgery	1450 (6.1)	782 (6.4)	537 (5.9)	131 (5.6)	0.17
Percutaneous coronary intervention	2346 (9.9)	1413 (11.6)	725 (8.0)	208 (8.9)	<0.001
Ischemic stroke	1235 (5.2)	535 (4.4)	599 (6.6)	101 (4.3)	<0.001
Cerebrovascular disease	4990 (21.1)	2419 (19.8)	2093 (23.0)	478 (20.4)	<0.001
Other thromboembolic event	191 (0.8)	79 (0.6)	96 (1.1)	16 (0.7)	0.003
Ventricular tachycardia or fibrillation	751 (3.2)	354 (2.9)	333 (3.7)	64 (2.7)	0.003
Mitral and/or aortic valvular disease	5928 (25.1)	2318 (19.0)	3010 (33.1)	600 (25.6)	<0.001
Peripheral arterial disease	2001 (8.5)	985 (8.1)	836 (9.2)	180 (7.7)	0.004
Rheumatic heart disease	585 (2.5)	212 (1.7)	327 (3.6)	46 (2.0)	<0.001
Cardiac resynchronization therapy	54 (0.2)	18 (0.1)	26 (0.3)	10 (0.4)	0.01
Implantable‐cardioverter defibrillator	806 (3.4)	418 (3.4)	313 (3.4)	75 (3.2)	0.83
Pacemaker	1648 (7.0)	588 (4.8)	931 (10.3)	129 (5.5)	<0.001
Dyslipidemia	15 943 (67.4)	8499 (69.6)	5835 (64.3)	1609 (68.5)	<0.001
Hypertension	18 735 (79.2)	9597 (78.6)	7279 (80.2)	1859 (79.2)	0.02
Diabetes mellitus	5694 (24.1)	2961 (24.2)	2163 (23.8)	570 (24.3)	0.76
Hospitalized bleed	1578 (6.7)	655 (5.4)	785 (8.6)	138 (5.9)	<0.001
Diagnosed dementia	1787 (7.6)	853 (7.0)	796 (8.8)	138 (5.9)	<0.001
Diagnosed depression	4439 (18.8)	2459 (20.1)	1583 (17.4)	397 (16.9)	<0.001
Chronic lung disease	9904 (41.9)	4992 (40.9)	3961 (43.6)	951 (40.5)	<0.001
Chronic liver disease	925 (3.9)	497 (4.1)	346 (3.8)	82 (3.5)	0.34
Mechanical fall	796 (3.4)	364 (3.0)	367 (4.0)	65 (2.8)	<0.001
Systemic cancer	1780 (7.5)	894 (7.3)	736 (8.1)	150 (6.4)	0.009
Baseline laboratory characteristics, mean (SD)					
eGFR, mL/min per 1.73 m^2^	59.6 (22.9)	61.3 (24.4)	57.7 (20.8)	58.5 (21.0)	<0.001
Hemoglobin, g/L	13.1 (1.9)	13.1 (1.9)	13.1 (1.8)	13.2 (1.9)	0.007
Systolic blood pressure, mm Hg	130.7 (19.2)	132.1 (19.9)	128.6 (18.1)	131.9 (18.6)	<0.001
Diastolic blood pressure, mm Hg	75.8 (11.3)	76.2 (11.5)	75.2 (11.0)	76.6 (10.8)	<0.001
HDL, g/dL	47.7 (14.8)	47.4 (14.5)	47.9 (15.0)	48.2 (14.9)	0.03
LDL, g/dL	96.7 (33.7)	99.2 (35.1)	93.4 (31.7)	96.8 (32.5)	<0.001

AF indicates atrial fibrillation; eGFR, estimated glomerular filtration rate; HDL, high‐density lipoprotein; LDL, low‐density lipoprotein.

**Table 2.** Baseline Medication Use Among 23 644 Adults With Heart Failure and Preserved or Reduced Left Ventricular Systolic Function Identified During 2005–2008, Overall and Stratified by AF Status

Baseline Medication Use*	Overall (N=23 644)	No AF (N=12 215)	Preexisting AF (N=9081)	Incident AF (N=2348)	*P*‐Value
ACEI/ARB	13 686 (57.9)	7206 (59.0)	5099 (56.2)	1381 (58.8)	<0.001
Aldosterone receptor antagonist	1963 (8.3)	997 (8.2)	770 (8.5)	196 (8.3)	0.71
β‐Blocker	14 807 (62.6)	7411 (60.7)	6000 (66.1)	1396 (59.5)	<0.001
Calcium channel blocker	6089 (25.8)	2911 (23.8)	2596 (28.6)	582 (24.8)	<0.001
Digoxin	4115 (17.4)	1107 (9.1)	2732 (30.1)	276 (11.8)	<0.001
Diuretic (loop)	11 969 (50.6)	5771 (47.2)	5018 (55.3)	1180 (50.3)	<0.001
Diuretic (thiazide)	4257 (18.0)	2227 (18.2)	1565 (17.2)	465 (19.8)	0.01
Nitrate	4591 (19.4)	2532 (20.7)	1566 (17.2)	493 (21.0)	<0.001
Statin	12 528 (53.0)	6758 (55.3)	4486 (49.4)	1284 (54.7)	<0.001
Other lipid‐lowering drug	1377 (5.8)	766 (6.3)	461 (5.1)	150 (6.4)	0.001
Antiplatelet agent	2237 (9.5)	1416 (11.6)	587 (6.5)	234 (10.0)	<0.001
Anticoagulant	5555 (23.5)	751 (6.1)	4545 (50.0)	259 (11.0)	<0.001
Statin	12 528 (53.0)	6758 (55.3)	4486 (49.4)	1284 (54.7)	<0.001
Other lipid‐lowering drug	1377 (5.8)	766 (6.3)	461 (5.1)	150 (6.4)	0.001

AF indicates atrial fibrillation; ACEI, angiotensin‐converting enzyme inhibitor; ARB, angiotensin receptor blocker.

* From 120 days before index date.

### AF and Death From Any Cause

Median follow‐up of the overall cohort was 1.8 years (interquartile range 0.8 to 3.1). The rate of death from any cause in the overall cohort was 14.1 per 100 person‐years (95% CI 13.8 to 14.5). The crude rate (per 100 person‐years) of death was higher in those with incident AF compared with those who had preexisting AF or no AF (Table 3). In the overall cohort, after adjustment for potential confounders, compared with those who did not have AF, incident and preexisting AF was associated with a higher risk of death, with adjusted hazard ratios (HRs) of 1.67 (95% CI 1.52 to 1.84) and 1.13 (95% CI 1.07 to 1.20), respectively. Similar findings were found in those with HF‐REF versus HF‐PEF (Table 4). Further adjustment for longitudinal use of medications (eg, angiotensin‐converting enzyme inhibitors, angiotensin receptor blockers, aldosterone antagonists, β‐blockers, calcium channel blockers, digoxin, thiazide and loop diuretics, nitrates, statins, other lipid‐lowering therapies, anticoagulants, and antiplatelet agents) during follow‐up did not significantly alter the observed associations.

**Table 3.** Crude and Adjusted Rates for Ischemic Stroke, Hospitalization for Heart Failure, Hospitalization for Any Cause, Death From Any Cause Among 23 644 Patients With Heart Failure, Overall and Stratified by AF Status

Variable	n (%)	Total Person‐years	Mean Person‐years (SD)	Rate per 100 Person‐years (95% CI)	Rate per 100 Person‐years, Adjusted for Age and Sex (95% CI)
Crude and adjusted rates to death from any cause					
Overall	6394 (27.0)	45 313.7	1.9 (1.3)	14.1 (13.8 to 14.5)	14.1 (14.1 to 14.2)
No AF	2866 (19.4)	25 431.3	1.7 (1.3)	11.3 (10.9 to 11.7)	12.4 (12.4 to 12.5)
Preexisting AF	2853 (31.4)	16 710.8	1.8 (1.3)	17.1 (16.4 to 17.7)	15.2 (15.1 to 15.2)
Incident AF	675 (28.8)	3178.1	1.4 (1.1)	21.2 (19.6 to 22.8)	20.3 (20.2 to 20.4)
Crude and adjusted rates to first hospitalization for heart failure					
Overall	6273 (26.5)	37 763.3	1.5 (1.3)	16.6 (16.2 to 17.0)	16.6 (16.5 to 16.7)
No AF	3233 (22.2)	21 741.6	1.5 (1.3)	14.9 (14.4 to 15.4)	15.4 (15.3 to 15.4)
Preexisting AF	2598 (28.6)	13 766.0	1.5 (1.3)	18.9 (18.2 to 19.6)	17.7 (17.7 to 17.8)
Incident AF	442 (23.9)	2260.8	1.2 (1.1)	19.6 (17.7 to 21.4)	19.1 (19.0 to 19.2)
Crude and adjusted rates to first hospitalization for any cause					
Overall	15 744 (66.6)	23 099.1	1.0 (1.1)	68.2 (67.1 to 69.2)	68.2 (67.4 to 68.9)
No AF	8795 (60.4)	13 944.9	1.0 (1.1)	63.1 (61.8 to 64.4)	65.0 (64.3 to 65.6)
Preexisting AF	6224 (68.5)	8261.9	0.9 (1.0)	75.3 (73.5 to 77.2)	71.8 (71.0 to 72.6)
Incident AF	725 (64.7)	895.3	0.8 (0.9)	81.0 (75.1 to 86.9)	78.1 (77.2 to 79.0)
Crude and adjusted rates to first ischemic stroke					
Overall	906 (3.8)	44 531.3	1.9 (1.3)	2.0 (1.9 to 2.2)	2.0 (2.0 to 2.0)
No AF	385 (2.6)	25 079.0	1.7 (1.3)	1.5 (1.4 to 1.7)	1.6 (1.6 to 1.6)
Preexisting AF	417 (4.6)	16 402.1	1.8 (1.3)	2.5 (2.3 to 2.8)	2.3 (2.3 to 2.3)
Incident AF	104 (4.5)	3056.5	1.3 (1.1)	3.4 (2.8 to 4.1)	3.5 (3.5 to 3.5)

AF indicates atrial fibrillation.

**Table 4.** Association Between AF and Death From Any Cause, Hospitalization for Heart Failure, Hospitalization for Any Cause, and Ischemic Stroke Among 24 175 Adults With Heart Failure, Overall and Stratified by Preserved and Reduced Ventricular Systolic Function (2005–2008)

AF Status	Overall (N=24 175)	Preserved Systolic Function (n=14 295)	Reduced Systolic Function (n=9880)
Death from any cause, adjusted* hazard ratio (95% CI)			
No AF	Reference	Reference	Reference
Preexisting AF	1.13 (1.07 to 1.20)	1.11 (1.03 to 1.20)	1.15 (1.05 to 1.26)
Incident AF	1.67 (1.52 to 1.84)	1.62 (1.42 to 1.84)	1.72 (1.48 to 1.98)
Hospitalization for heart failure, adjusted* hazard ratio (95% CI)			
No AF	Reference	Reference	Reference
Preexisting AF	1.22 (1.15 to 1.29)	1.26 (1.17 to 1.37)	1.16 (1.05 to 1.27)
Incident AF	2.00 (1.83 to 2.18)	1.96 (1.73 to 2.22)	2.04 (1.80 to 2.31)
Hospitalization for any cause, adjusted* hazard ratio (95% CI)			
No AF	Reference	Reference	Reference
Preexisting AF	1.15 (1.11 to 1.19)	1.16 (1.11 to 1.21)	1.12 (1.06 to 1.18)
Incident AF	1.45 (1.37 to 1.54)	1.43 (1.33 to 1.54)	1.49 (1.37 to 1.63)
Ischemic stroke, adjusted* hazard ratio (95% CI)			
No AF	Reference	Reference	Reference
Preexisting AF	1.57 (1.34 to 1.83)	1.91 (1.56 to 2.33)	1.07 (0.82 to 1.39)
Incident AF	2.47 (1.97 to 3.09)	2.72 (2.05 to 3.61)	2.16 (1.48 to 3.14)

AF indicates atrial fibrillation; GFR, glomerular filtration rate; HDL, high‐density lipoprotein; LDL, low‐density lipoprotein.

* All models were also adjusted for age, sex, left ventricular ejection fraction (in overall models only), prevalent heart failure, acute myocardial infarction, unstable angina, coronary artery bypass graft surgery, percutaneous coronary intervention, ischemic stroke, other thromboembolic event, ventricular fibrillation or ventricular tachycardia, peripheral arterial disease, cardiac resynchronization therapy, implantable‐cardioverter defibrillator, pacemaker, dyslipidemia, hypertension, diabetes mellitus, hospitalized bleeds, diagnosed dementia, diagnosed depression, chronic lung disease, chronic liver disease, mechanical fall, systemic cancer, estimated GFR, hemoglobin, systolic blood pressure, HDL cholesterol, LDL cholesterol, race, and site.

* Ischemic stroke outcome models were adjusted for age, sex, left ventricular ejection fraction (in overall models only), prevalent heart failure, acute myocardial infarction, unstable angina, coronary artery bypass graft surgery, percutaneous coronary intervention, prevalent ischemic stroke, other thromboembolic event, ventricular fibrillation or ventricular tachycardia, peripheral arterial disease, cardiac resynchronization therapy, implantable cardioverter defibrillator, pacemaker, dyslipidemia, hypertension, diabetes mellitus, hospitalized bleeds, diagnosed dementia, diagnosed depression, chronic lung disease, chronic liver disease, mechanical fall, systemic cancer, estimated GFR, hemoglobin, systolic blood pressure, HDL cholesterol, LDL cholesterol, race, and site.

### AF and Ischemic Stroke

The rate of ischemic stroke was 2.0 per 100 person‐years (95% CI 1.9 to 2.2) in the overall cohort. Crude rates (per 100 person‐years) of ischemic stroke were 1.5, 2.5, and 3.4 for those with no AF, preexisting AF, and incident AF, respectively (Table 3). In the overall cohort, after adjustment for potential confounders, compared with patients who did not have AF, preexisting AF and incident AF were associated with a higher risk of ischemic stroke, with HR 1.57 (95% CI 1.34 to 1.83) and 2.47 (95% CI 1.97 to 3.09), respectively (Table 4). Incident AF was associated with a 2‐fold higher HR of ischemic stroke in patients with HF‐PEF as well as HF‐REF, but preexisting AF was associated with ischemic stroke only in those with HF‐PEF (Table 4). Additional adjustment for longitudinal cardiovascular medication use did not materially change the results.

### AF and Hospitalization for HF

In the overall cohort, the rate of hospitalization for HF was 16.6 per 100 person‐years (95% CI 16.2 to 17.0). Crude rates (per 100 person‐years) were higher in those with preexisting AF (18.9) or with incident AF (19.6) compared with patients who did not have AF (14.9) (Table 3). In the overall cohort, after adjustment for several potential confounders, compared with patients who did not have AF, preexisting AF was associated with a higher adjusted HR of hospitalization for HF (HR 1.22, 95% CI 1.15 to 1.29), and incident AF was associated with a 2‐fold higher adjusted HR of hospitalization for HF (HR 2.00, 95% CI 1.83 to 2.18), with similar results in those with HF‐PEF or HF‐REF (Table 4). Further adjustment for longitudinal medication use did not alter findings appreciably (Table 4).

### AF and Hospitalization From Any Cause

The rate of hospitalization from any cause in the overall cohort was 68.2 per 100 person‐years (95% CI 67.1 to 69.2). Crude rates (per 100 person‐years) were higher in those with preexisting AF (75.3) or incident AF (81.0) compared with patients who did not have AF (63.1) (Table 3). In the overall cohort, after adjustment for potential confounders, compared with patients who did not have AF, preexisting and incident AF were associated with higher risk of hospitalization from any cause, with HR of 1.15 (95% CI 1.11 to 1.19) and HR of 1.45 (1.37 to 1.54), respectively, and similar results in those with HF‐PEF or HF‐REF (Table 4). Additional adjustment for longitudinal medication use did not materially affect the results.

## Discussion

Within a multiethnic community‐based cohort of >23 600 adults with HF, we demonstrate that both preexisting and incident AF are common in patients with HF and are associated with major adverse cardiovascular outcomes. Newly diagnosed AF complicating HF was associated with the highest risk for adverse complications, but the higher multivariable adjusted hazards of ischemic stroke, hospitalization for HF, hospitalization for any cause, and death associated with AF did not materially differ between participants with HF‐PEF and those with HF‐REF. These associations persisted even after accounting for a broad spectrum of potential confounders as well as differential longitudinal exposure to relevant medications.

We found that nearly one third of patients with HF‐REF had preexisting AF, which is consistent with previous estimates (15% to 35%).^20–23^ Although data are more limited for patients with HF‐PEF, the frequency of preexisting AF (43.2%) among patients with HF‐PEF with our study was similar to the proportion of patients with AF in the Irbesartan in heart failure with PRESERVEd systolic function (I‐PRESERVE) study as well as 2 studies involving 2802 (31.8%) and 6072 (41.3%) subjects with HF conducted in 2001.^24^ We also noted a high and relatively similar rate of new‐onset AF complicating HF‐REF and HF‐PEF, with approximately 1 in 10 participants developing AF during a median 1.8 years of follow‐up. Incidence rates of AF observed in our study are significantly higher than those reported in 2 community‐based investigations involving 708 and 1664 patients with HF, in which 17% and 23% of patients with HF developed new‐onset AF during 4 years of follow‐up, respectively.^7^ The higher rate of incident AF in our study likely relates to the higher proportion of subjects with HF‐PEF, higher prevalence of AF risk factors, including hypertension and diabetes, and access to ambulatory diagnosis data, compared with other studies.

The epidemiologic similarities between AF and HF, as well as their frequent concurrence, are explained, at least in part, by shared underlying risk factors, including hypertension, diabetes mellitus, chronic kidney disease, ischemic heart disease, and valvular heart disease.^25^ As expected, we found that patients with HF with AF in our cohort were more likely to have worse kidney function, documented hypertension, and known valvular heart disease.^26^ Notably, however, patients with HF and AF were not more likely to have diabetes or coronary artery disease than were patients without AF, which is consistent with other studies.^27–28^

Absolute rates of death in our study were high and consistent with rates seen in previous studies of patients with AF and HF. Among 1470 individuals with HF from the Framingham Heart Study, incident AF was associated with a 60% higher risk for death in men and nearly 3‐fold higher risk for death in women.^7^ Among participants in the Candesartan in Heart failure—Assessment of Reduction in Mortality and morbidity (CHARM) study, crude death rates were 24% in those with AF and HF‐PEF and 37% in those with HF‐REF.^28^ In contrast to CHARM,^28^ in our study of 23 644 participants with HF, we found that incident AF was associated with a notably higher rate of death that was similar in those with HF‐PEF or HF‐REF. The reasons for the differences between our study and CHARM are not clear but may relate to differences between the selected subjects with HF enrolled in the CHARM clinical trial versus the more representative patients with HF treated in community‐based practice settings in our study.

As expected, we found that AF was associated with an elevated risk for ischemic stroke in adults with HF. We observed that incident AF conveyed a particularly high hazard of ischemic stroke (≈2.5‐fold higher adjusted rates compared with no AF), which is consistent with previous analyses.^29^ Of note, however, preexisting AF was associated with higher adjusted hazard of ischemic stroke in those with HF‐PEF but not for those with HF‐REF. Although the CHARM investigators reported higher rates of ischemic stroke in patients with known AF and HF‐PEF compared with HF‐REF (9% versus 6%, respectively), this difference did not achieve statistical significance (*P*=0.32).^28^ However, the CHARM study was underpowered to evaluate the association between prevalent AF and ischemic stroke due to a low number of fatal or nonfatal strokes in their cohort with AF (total n=28). The reasons for why preexisting AF was a strong predictor of ischemic stroke in those with HF‐PEF but not HF‐REF are not clear, but patients with preexisting AF may have preferentially experienced stroke or other AF‐related complications and death before the development of HF and therefore led to a more selected subgroup of patients with preexisting AF and HF in our cohort. Although we adjusted for age, the interaction between age and risk for ischemic stroke might have modified the relation between preexisting AF and HF‐PEF in a manner distinct from HF‐REF. Moreover, although we did not observe significant differences in the death rates of patients with HF‐PEF compared with HF‐REF, it is also possible that our findings could also be explained by the fact that patients with HF‐REF may have been more likely than patients with HF‐PEF to die before ischemic stroke develops.

Overall rates of hospitalization observed in our study (66.6%) were higher than those reported in a study of 17 448 Medicare beneficiaries hospitalized for HF (44% rehospitalized in the 6 months after discharge) during 1991–1994.^30^ In contrast to the findings of a Japanese HF registry, which included 319 patients with HF hospitalized in 2006–2007,^31^ we observed a significant increase in adjusted rates of HF‐related and all‐cause hospitalization among patients with preexisting and incident AF. Our findings are consistent with those of the CHARM substudy, which found a 4‐fold higher risk for cardiovascular‐related rehospitalization in patients with new‐onset AF and HF, regardless of systolic function.^28^ Even after extensive adjustment for potential confounders and longitudinal use of therapies, we found an approximately 1.5‐fold higher hazard of hospitalization associated with incident AF. The difference in the magnitude of association between these findings may be explained by the greater comorbid disease burden in our community‐based cohort compared with the younger and healthier participants enrolled in the CHARM clinical trial. This hypothesis is supported by the overall higher rates of HF and all‐cause hospitalizations in our study relative to CHARM. Our finding that AF had a similarly negative impact on HF‐specific and all‐cause hospitalizations among patients with HF‐PEF and those with HF‐REF highlight the importance of AF on the high burden and cost to patients and the health care system.

Our study included a large socioeconomically and racially diverse multicenter cohort recruited from multiple geographic areas and varying clinical practice settings. Another strength of our study is the use of a standardized data resource (CVRN VDW) with linked demographic, health care utilization, pharmacy, laboratory, and vital status information. Our study also had several limitations. Our study was conducted in an insured population, so the findings may not be fully generalizable to uninsured persons or other practice settings. The large sample size facilitated many statistically significant findings, but we focused on clinically meaningful effect sizes. Information was unavailable on the type of AF (eg, paroxysmal, persistent, permanent), although previous studies support similar relationships between AF type and the risk of ischemic stroke.^32^ We did not include information about certain AF treatment modalities, including AF ablation, but prior investigations have not consistently shown that AF ablation reduces HF hospitalizations, total death rates, or the risk of stroke.^33^ Because we relied on clinically obtained assessments of left ventricular systolic function, systematic data on structural aspects of the atria were unavailable. However, we believe that our sample represents a “real world” cohort of adults with clinically recognized HF managed in typical care settings and therefore provides generalizable results to the broader US population.

In sum, both preexisting and new‐onset AF were frequent complications of HF and increased the rates of ischemic stroke, hospitalization for HF or any cause, and death, overall and similarly in those with HF‐PEF or HF‐REF. Incident AF consistently carried a worse prognosis for each of these outcomes compared with preexisting AF. Our study emphasizes the need to develop novel prevention strategies for AF and its associated complications in patients with HF‐PEF and HF‐REF.
